# The effects of organic waste materials on *Miscanthus × giganteus* yield and Zn and Ni content

**DOI:** 10.1038/s41598-024-67413-y

**Published:** 2024-07-16

**Authors:** Elżbieta Malinowska, Paweł Kania

**Affiliations:** Faculty of Agricultural Sciences, University of Siedlce, Stanisława Konarskiego 2 Str., 08-110 Siedlce, Poland

**Keywords:** *Miscanthus × giganteus*, Biomass yield, Organic waste, Zn, Ni, Rhizomes, Soil, Plant ecology, Energy science and technology

## Abstract

The aim of the experiment was to determine the yield of *Miscanthus × giganteus* M 19 in the first three years of cultivation and its bioaccumulation of Zn and Ni in aboveground and underground parts in response to different doses of sewage sludge and substrate left after the production of white mushrooms. *Miscanthus × giganteus* is a grass species that adapts to different environmental conditions and can be grown in various climatic zones of Europe and North America. In April 2018 the experiment was established in a randomized block design and with four replications in central-eastern Poland*.* Waste organic materials (municipal sewage sludge and mushroom substrate) were applied to the soil in 2018 in the spring before the rhizomes of giant miscanthus were planted. Each year (from 2018 to 2020) biomass was harvested in December. The yield of fresh and dry matter and the total content of Zn and Ni, after wet mineralization of plant samples, were determined by optical emission spectrometry (ICP-OES). After the third year of cultivation, the content of Zn and Ni in rhizomes and in the soil was determined again. In relation to control, an increase in the yield of miscanthus biomass in response to organic waste materials was noted. Plants responded to mushroom substrate (SMS) with the highest average yield (16.89 Mgha^−1^DM), while on the control plot it was 13.86 Mg  ha^−1^DM. After the third year of cultivation, rhizomes of *Miscanthus x giganteus* contained higher amounts of Zn (63.3 mg kg^−1^) and Ni (7.54 mg kg^−1^) than aboveground parts (40.52 and 2.07 mg kg^–1^), which indicated that heavy metals were retained in underground parts.

## Introduction

With its C4 type of photosynthesis, *Miscanthus × giganteus* produces biomass that is increasingly used as a renewable fuel^1–4^. Even if economically and ecologically it is a very promising plant, there are still challenges to its widespread commercial role. The cultivation of energy crops is a controversial issue because they compete with plants grown for food production. Therefore, giant miscanthus should be grown in areas excluded from the production of crops intended for food and animal feed, for example, near motorways and roads with high traffic intensity and on marginal and degraded soils^5^. Due to its high biomass yield per unit area *Miscanthus* × *giganteus* requires much less acreage than low-yielding crops^3^. To produce the same amount of biomass as other energy grass species, giant miscanthus requires 87% less land^3^. Perennial energy crops produce yields equal to or better than annual crops. With good environmental performance, they improve ecosystem health for many years and are compatible with sustainable development. Perennial energy grasses protect soil from wind and water erosion, while improving its quality, especially if organic waste materials of a fertilizing nature are used^6–8^. A potential increased share of perennials is also associated with an increase in landscape biodiversity as such plants provide habitat for wild animals and insects^9,10^. Organic waste such as municipal sewage sludge, mushroom substrate, distillers grains or digestate from biogas plants can be successfully used in *Miscanthus × giganteus* cultivation. They improve the physicochemical properties of soils, especially poor ones^11–13^, positively affecting the growth of various plants^6,14^.

In Poland, due to the very rapid development of mushroom farms, large amounts of spent mushroom substrate are generated so it is necessary to properly manage it in order to avoid its adverse impact on the natural environment. Municipal sewage sludge generated as a result of wastewater treatment is an organic waste that requires appropriate management. Due to economic development, improved living standards and legal requirements, the current trend of increasing the amount of municipal sewage sludge will continue. According to many authors^15,16^, the application of sewage sludge to soil enriches it with nutrients and organic matter. However, the main barrier limiting its use is the excessive content of heavy metals and the presence of pathogenic organisms. Most heavy metals are easily accumulated by plants^17^.

The aim of the experiment was to determine the yield of *Miscanthus × giganteus* treated with municipal sewage sludge and mushroom substrate during the first three years of cultivation in central-eastern Poland. Another objective was to assess Zn and Ni soil content and their bioaccumulation in the aboveground and underground parts of plants.

## Methods

### Description of the experiment

The field experiment was established on the experimental facility of Siedlce University (52^o^17'N, 22^o^28′ E), on compact sandy loam soil, with loose sandy loam as subsoil, of the anthropogenic order, cultured type and hortisol subtype^18^. The experiment was conducted in a randomized block design, with four replications. The plant used in the studies was *Miscanthus × giganteus* M 19, a perennial grass of the C4 photosynthetic type. At the end of April 2018, its rhizomes were cut into pieces and manually planted on microplots with an area of 2 m^2^, to a depth of 20 cm. The seedlings of *Miscanthus × giganteus* were previously purchased in a plant nursery. Per one square meter three pieces were planted, all 8–10 cm long and with 3–4 buds. The distance between the seedlings was 30 cm. The planting rate per 1 ha was about 30 thousand pieces of rhizomes. The distance between the plots was 1 m.

The experiment was established according to the following scheme:

**Table:** 

Symbols	Experimental plots
Control	Control plot (no treatment)
(SS)	Municipal sewage sludge—170 kg N ha^−1^ (0.907 kg per plot, or 4.54 Mg ha^−1^);
(SS_75_ + SMS_25_)	municipal sewage sludge + mushroom substrate—170 kg N ha^−1^ with75% of SS + 25% of SMS (0.683 kg + 1.40 kg per plot, or 3.38 Mg ha^−1^ + 7 Mg ha^−1^)
(SS_50_ + SMS_50_)	municipal sewage sludge + mushroom substrate—170 kg N ha^−1^ with 50% of SS + 50% of SMS (0.454 kg + 2.80 kg per plot, or 2.25 Mg ha^−1^ + 14 Mg ha^−1^)
(SS_25_ + SMS_25_)	municipal sewage sludge + mushroom substrate—170 kg N ha^−1^ with 25%of SS + 75% of SMS (0.227 kg + 4.20 kg per plot, or 1.13 Mg ha ^1^ + 21 Mg ha^−1^)
(SMS)	mushroom substrate-170 kg N ha^−1^ (5.60 kg per plot, or 28 Mg ha^−1^)

Organic waste materials (municipal sewage sludge and mushroom substrate) were applied once in the spring of 2018 before rhizomes were planted. They were mixed with soil to a depth of 25 cm. Municipal sewage sludge was provided by the sewage treatment plant in Siedlce, with its capacity of approx. 24 000 m^3^ per day. Annually, it produces 1 897 Mg of sewage sludge, an average of 5.2 Mg per day. Mushroom substrate was obtained from a mushroom farm located in the Siedlce district. The producer of the substrate for mushroom cultivation was Unikost, while peat moss for the casing layer was produced by Wokas. The plants took root on all experimental plots. Weeds were controlled mechanically, but not in the third year when hardly any of them were found. The dose of 170 kg ha^−1^ of N in an organic form was established according to the recommendations of the Nitrates Directive, aimed at reducing water pollution by nitrates from agricultural sources. Pollution prevention measures adopted by the Regulation of the Polish Council of Ministers^19^ were also taken into account.

### Determination of soil and organic material properties

The following were determined in air-dry soil samples collected from three layers: 0–20 cm, 20–40 cm and 40–60 cm before the experiment started and in those collected only from the arable layer (0–20 cm) in the third year:

- granulometric composition using the Bouyoucos-Casagrande hydrometric method modified by Prószyński in accordance with the Polish Standard PN-R-04033^20^ and with the classification according to the grain size of soil and of mineral formations^21^,
- pH value in H_2_O and in 1 mol l^−1^ of KCl by the potentiometric method,
- total nitrogen (N_t_) and carbon (C_t_) by elemental analysis using the PerkinElmer® 2400 Series II CHNS/O Elemental Analyzer with a thermal conductivity detector (TCD);
- content of total P, K, Zn and Ni (after wet mineralization of soil material with aqua regia) by optical emission spectrometry (ICP-OES) at Eurofins Environment Testing Poland Ltd. in Katowice, formerly the Centre for Environmental Research and Control,
- available forms of P and K by the Egner-Riehm method at the Regional Chemical-Agricultural Station in Lublin according to the following Polish Standards: PN-R-04023:1996 and PN-R-04022:1996+Az1:2004,
- available forms of Mg by the Schachtschabel method at the District Chemical and Agricultural Station in Lublin according to the PN-R-0420:1994+Az1:2004 Polish Standard.

In organic materials, the following were determined:

- dry matter content, after drying the sample at 105 °C until a constant weight;
- pH value in H2O and 1 mol l^−1^ of KCl by the potentiometric method;
- content of total nitrogen (Nt) by the modified Kjeldahl method, with mineralization of samples in concentrated sulphuric acid (VI) in the presence of a selenium mixture^22^;
- organic carbon by the oxidation-titration method^23^;
- total content of P and K as well as Zn and Ni by optical emission spectrometry (ICP-OES) after mineralization of samples with aqua regia.

The experiment was conducted on pH-neutral soil with high content of total C and N (Table 1). The content of Ni was several times lower than the amounts provided by the Regulations of the Ministry of Climate and the Environment^24^ for light soils treated with municipal sewage sludge. The content of Zn was within the permissible limit. The content of available forms of P, K and Mg in the top layer of soil was high (mg kg^−1^ of soil), with P_2_O_5_—117,K_2_O—47.5 and Mg—10.04. The soil and subsoil consisted of loamy sand, with 81% of sand, 16% of silt and 3% of clay in the arable-humus layer.

**Table 1:** Soil chemical properties before the experiment.

Layer of the soil profile (cm)	pH_H2O_	pH_KCl_	C_t_	N_t_	C:N	P	K	Zn	Ni
			(g kg^−1^)			(g kg^−1^)		(mg kg^−1^)	
0–20 (A1)	6.93	6.60	40.50	2.85	14.21	1.19	0.736	149.7	5.53
20–40 (A2)	6.25	5.85	20.30	1.65	12.30	0.784	0.680	136.1	4.29
40–60 (A3)	6.10	5.50	19.15	1.60	11.97	0.581	0.502	46.59	2.27

### Determination of fresh and dry matter yield

The biomass of giant miscanthus was harvested in December 2018 (first year), 2019 (second year) and 2020 (third year). Then the fresh matter was weighed to determine the yield. A representative sample of plants with five leafy shoots was collected from each plot, and dry matter content was determined by drying it at 105 °C until a constant weight was obtained. The plants were not rinsed before chemical analyses. After sample preparation, i.e. after shredding and grinding it, the total content of Zn and Ni was determined by emission optical spectrometry (ICP-OES) after wet mineralization of the samples with aqua regia. Additionally, at the end of the third growing period, rhizomes were collected in order to determine their content of Zn and Ni.

### Meteorological conditions

Meteorological data for three years of research (2018–2020) were provided by the Institute of Meteorology and Water Management, National Research Institute (PIB) in Warsaw. In order to interpret the results more accurately, Selyaninov’s hydrothermal coefficient(K) was calculated, expressing the temporal variability of precipitation and of air temperature, according to the following formula:

$$K = \frac{P}{{0.1{\Sigma t}}}$$

where:

P—monthly precipitation,

Σt—the sum of daily air temperature values in a given month^25^.

**Figure 1:**
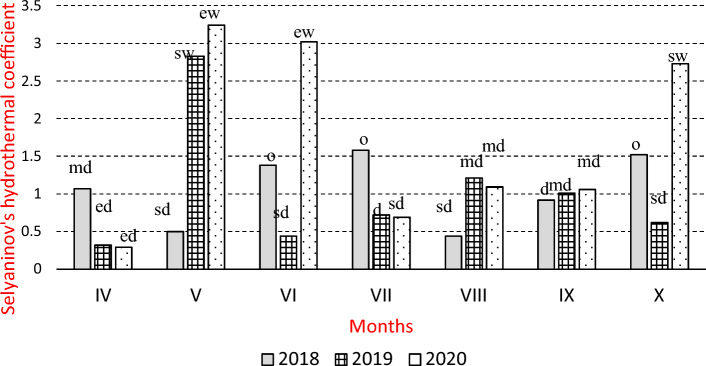
Selyaninov’s hydrothermal coefficient (K) during the growing periods of 2018–2020.

The values of Selyaninov’s hydrothermal coefficient (K) were grouped into the following ranges:

K ≤ 0.4 extreme drought (ed),

0.4 < K ≤ 0.7 severe drought (sd),

0.7 < K ≤ 1.0 drought (d),

1.0 < K ≤ 1.3 moderate drought (md),

1.3 < K ≤ 1.6 optimal (o),

1.6 < K ≤ 2.0 moderately wet (mw),

2.0 < K ≤ 2.5 wet (w),

2.5 < K ≤ 3.0 severely wet (sw),

K > 3.0 extremely wet (ew)^26^ (Fig. 1)

According to Sielyaninov’s coefficient (K), optimal thermal and moisture conditions were only in June, July and October of 2018 (Fig. 1). However, in June of 2019 and of 2020 extreme weather conditions were recorded, and May and June 2020 were extremely wet. The most unfavourable conditions for miscanthus growth were in 2019, as they ranged from extremely dry to quite dry in each month, with the exception of May. According to Danalatos et al.^27^, the daily growth of giant miscanthus shoots is the most intense from May to the beginning of June and may amount to 3 cm, therefore, unfavourable meteorological conditions in this period may limit yields.

### Ethical approval

The authors hereby declare that the methods were carried out in accordance with relevant guidelines.

### Result processing

The results were statistically developed using the analysis of variance for a two-factor experiment. The significance of the effect of experimental factors on feature values was determined with the F Fisher-Snedecor test. The value of LSD_0.05_ (for a detailed comparison of means) was calculated with Tukey’s test, and StatisticaStatSoft 13.1^28^ was used for calculations.

The uptake of Zn and Ni by *Miscanthus × giganteus* was calculated based on their total content in aboveground dry matter yield. The bioaccumulation coefficient (BC) of Zn and Ni was calculated as aratio between the average concentration of an element in the plant (C_shoot_)and in the soil C_soil_^29^.

$${\text{BC}}_{{{\text{shoot}}}} = {\text{ C}}_{{{\text{shoot}}}} :{\text{ C}}_{{{\text{soil}}}}$$

$${\text{BC}}_{{{\text{rhizome}}}} = {\text{ C}}_{{{\text{rhizome}}}} :{\text{ C}}_{{{\text{soil}}}}$$

BC_shoot_—coefficient of bioaccumulation of heavy metals in the aboveground parts of giant miscanthus; BC_rhizome_—coefficient of heavy metal bioaccumulation in giant miscanthus rhizomes.

The translocation factor (TF) of heavy metals (Zn and Ni) was calculated according to the following formula:

$${\text{TF }} = {\text{ C}}_{{{\text{shoot}}}} :{\text{ C}}_{{{\text{rhizome}}}}$$

where:

TF—heavy metal translocation factor; C_shoot_—content of heavy metals in the aboveground parts of miscanthus (mg kg^−1^); C_rhizome_—content of heavy metals in miscanthus rhizomes (mg kg^−1^); C_soil_—content of heavy metals in the soil (mg kg^−1^).

The Pearson linear correlation coefficient between the following variables (arranged in pairs) was calculated: total Zn and Ni content in the soil, in the aboveground parts of *Miscanthus × giganteus*, in its rhizomes, Zn and Ni content in biomass, bioaccumulation coefficient and translocation factor.

## Results and discussion

What determines the value of organic waste materials, apart from their nutrient content and yield-increasing potential, is the dynamics of their decomposition in soil^6^. As regards municipal sewage sludge, its excessive content of heavy metals and soil microbiological contamination may exclude its application to crops. However, when its composition indicates that it can be used in agriculture, it has manifold benefits even though its use is still at a low level. This is due to insufficient research on its properties and on its effects on soil, plants and the environment, but also because of reluctant social attitude. Municipal sewage sludge used in the present experiment contained high amounts of dry matter (93%),but also of N, with 40.50 g kg^−1^DM, and of P, with 19.81 g kg^−1^DM (Table 2). According to the literature^30^, its K amounts are low, which was also confirmed by the present experiment (2.56 g kg^−1^). Even though in the present research pH of municipal sewage sludge was slightly acidic (pH 6.4), its application to acidic and degraded soils does not contribute to their acidification.In Poland, according to the Regulation of the Ministry of Climate and the Environment of 2020^31^, the pH value of soil treated with municipal sewage sludge cannot be lower than 5.6.

**Table 2:** Chemical properties and dry matter content of organic waste materials.

Organic waste material	pH_H2O_	pH_KCl_	DM	C_org_	C:N	N	P	K	Zn	Ni
			(%)	(g kg^−1^)		(g kg^−1^)			(mg kg^−1^)	
Municipal sewage sludge (SS)	6.40	5.95	93.0	348	7.80	40.5	19.8	2.56	987.2	44.2
Mushroom substrate (SMS)	6.41	6.01	30.0	284	13.6	20.9	8.86	11.2	156.9	4.84

Mushroom substrate used in the experiment contained as much as 30% of dry matter (Table 2), with Jordan et al.^32^ reporting similar amounts. N concentration of 20.9 g kg^−1^ was also high, while organic C concentration, as an indicator of soil biological activity, was 284 g kg^−1^. The C:N ratio was 13.59, indicating considerable mineralization of mushroom substrate N compounds with release of its nutrients. The concentration of mushroom substrate P was 8.86 g kg^−1^, with 11.21 g kg^−1^ of K. In the organic waste materials, the content of Zn and Ni did not exceed the standards set by the Regulations of the Ministry of Climate and the Environment^24^, which meant that their application to *Miscanthus × giganteus* was allowed.

The fresh matter yield of giant miscanthus aboveground parts, as an average of treatment combinations and years of research, was 26.22 Mg ha^−1^ (Table 3). It varied significantly over growing periods and was the lowest in the first one. In subsequent years, the yields were higher. The low value in the first year might have been caused by the insufficient development of roots, necessary for plant growth^33,34^. The highest yield of 39.58 Mg ha^−1^FM, average across treatment combinations, was recorded in the third year. The biomass yield of giant miscanthus harvested in the third growing period was higher by 160% than in the first and by 60% in the second. Gubiśová et al.^35^ report that miscanthus reaches its full productivity in the third year after its plantation.

**Table 3:** The yield of *Miscanthus × giganteus* (Mg ha^−1^).

Years (B)	Experimental plots (A)						
	Control plot	SS	SS_75_ + SMS_25_	SS_50_ + SMS_50_	SS_25_ + SMS_75_	SMS	Mean
Fresh matter yield (Mg ha^−1^)							
2018 (I)	12.50cA	13.35cA	14.75cA	15.00cA	17.50cA	17.10cA	15.03c
2019 (II)	19.95bBC	24.80bABC	23.75bABC	21.05bBC	25.95bAB	28.50bA	24.05b
2020 (III)	36.00aB	43.10aA	41.65aA	38.80aAB	39.60aAB	38.35aAB	39.58a
Mean	22.82C	27.08AB	26.72AB	25.05BC	27.68AB	27.98A	26.22
Dry matter yield(Mg ha^−1^)							
2018 (I)	9.30Ab	9.75cA	10.16cA	11.07bA	11.65cA	10.87cA	10.47c
2019 (II)	11.78Aab	15.50bA	16.12bA	10.79bA	15.12bA	14.42bA	13.96b
2020 (III)	20.49Ba	22.24aAB	22.18aAB	21.13aB	18.05aB	25.38aA	21.58a
Mean	13.86B	15.83AB	16.15A	14.33AB	14.94AB	16.89A	15.33

Within a row, different uppercase letters indicate a significant difference, within a column, different lowercase letters indicate a significant difference.

SS—municipal sewage sludge dose introducing 170 kg Nha^−1^; SMS—mushroom substrate dose introducing 170 kg Nha^−1^; SS_75_ + SMS_25_; SS_50_ + SMS_50_; SS_25_ + SMS_75_—municipal sewage sludge used together with mushroom substrate in various proportions, each dose introducing 170 kg Nha^−1^.

The yield of *Miscanthus × giganteus* fresh matter significantly varied across treatment combinations and was considerably higher on fertilized plots than on the control one. In the first year the highest fresh matter yield of 17.50 Mg ha^−1^ was recorded on the plot where the lowest dose of sewage sludge in combination with the highest dose of mushroom substrate was applied (SS_25_ + SMS_75_). The next highest value of 17.10 Mg ha^−1^ was noted on the plot with mushroom substrate applied on its own (SMS), while the lowest yield was on the control plot (12.50 Mg ha^−1^). A similar tendency was observed in the second year, with the highest fresh matter yield in response to the application of mushroom substrate on its own and in combination with sewage sludge, with 28.50 and 25.95 Mg ha^−1^, respectively. In the third year, the highest value was recorded on the plot with municipal sewage sludge (43.10 Mg ha^−1^) and the lowest on the control plot (36.00 Mg ha^−1^).

Thus, *Miscanthus × giganteus* dry and fresh matter yields varied across treatment combinations, which can be explained by the fact that the plants on different plots varied in their proportion of leaves in relation to shoots. The share of leaves can be up to 38% on average, and their water absorption capacity is higher^34^.

In the third year, the average dry matter yield of *Miscanthus × giganteus*, with 21.58 Mg ha^−1^,was more than twice as high as in the first year. It significantly varied throughout the experiment due to a growing number of shoots and because plants were taller year by year, with Angelini et al.^36^ having observed the same. Compared to control, the organic materials used in the experiment had a significant effect on the average dry matter yield of giant miscanthus, except for the plot on which sewage sludge and mushroom substrate were applied together in equal doses (SS_50_ + SMS_50_), both containing the same amount of nitrogen. On average, for three years, plants responded to mushroom substrate (SMS)with the highest fresh and dry matter yields.

Clifton-Brown and Lewandowski^33^ and Keymer and Kent^37^ found that giant miscanthus yield was affected by temperature distribution during the growing period and by soil moisture, but to a smaller extent by fertilizer treatment, especially by nitrogen supply. In the present experiment optimal thermal and moisture conditions were in June, July and October 2018 (Fig. 1). In other months, weather conditions varied. In June 2019 and 2020 (the second and third years) extreme droughts were recorded. Extremely wet conditions were in May and June 2020. The most unfavourable conditions for giant miscanthus were in 2019, as they ranged from extremely dry to moderately dry in each month with the exception of May (Fig. 1).

Zn concentration in plant biomass, average across treatment combinations, was the highest (52.90 mg kg^−1^DM) in the first year (Table 4). In the third year of the experiment, it was twice as low (28.45 mg kg^−1^DM), because biomass yield was more than twice higher than in the first (Table 3). Bilandžija et al.^38^ report a slightly higher Zn concentration in the biomass of the same plant harvested in winter (55.2 mg kg^−1^DM). On the other hand, according to Bosiacki et al.^39^, it ranged from 39.04 to 41.97 mg kg^−1^DM depending on the growing season. Lower Zn content was noted by Kotecki^34^ in giant miscanthus harvested in October.

**Table 4:** Concentration of Zn in *Miscanthus × giganteus* biomass (mgkg^−1^DM).

Years (B)	Experimental plots (A)						
	Control plot	SS	SS_75_ + SMS_25_	SS_50_ + SMS_50_	SS_25_ + SMS_75_	SMS	mean
	Zn (mg kg^−1^DM)						
2018 (I)	44.70aC	45.70aC	43.79aC	59.42aB	42.50bC	81.30aA	52.90a
2019 (II)	38.11bB	39.10bB	32.01bC	30.98bC	49.70aA	51.31bA	40.20b
2020 (III)	28.20cA	34.15bA	30.12bA	28.75bA	29.30cA	20.15cB	28.45c
Mean	37.00BC	39.65B	35.34C	39.72B	40.50B	50.92A	40.52

Within a row, different uppercase letters indicate a significant difference, within a column, different lowercase letters indicate a significant difference.

SS—municipal sewage sludge dose introducing 170 kg N ha^−1^; SMS—mushroom substrate dose introducing 170 kg N ha^−1^; SS_75_ + SMS_25_; SS_50_ + SMS_50_; SS_25_ + SMS_75_—municipal sewage sludge used together with mushroom substrate in various proportions, each dose introducing 170 kg N ha^−1^.

According to Bosiacki’s^40^ studies on phytoextraction potential, *Miscanthus × giganteus* is not a Zn and Cu hyper accumulator. The literature has confirmed its tolerance to increased concentrations of heavy metals in soil^41^. According to the above authors, osier willow (*Salix viminalis* L.) has a much greater potential to accumulate heavy metals than giant miscanthus. In the present experiment the highest concentration of Zn was found in plants treated with mushroom substrate (SMS) in the first and second years of cultivation, with 81.30 and 51.31 mg kg^−1^DM, respectively. On the other hand, its lowest amount (35.34 mg kg^−1^DM), as an average of all growing periods, was in plants treated with the highest dose of sewage sludge together with the lowest dose of mushroom substrate (SS_75_ + SMS_25_).

Across treatment combinations, the highest average Ni concentration (2.64 mg^.^kg^-1^DM) was in the aboveground parts of giant miscanthus in the first year (Table 5). Its decrease was noted in subsequent years of the experiment, due to its dilution with biomass yield that increased with the age of the plants (Table 3). The average concentration of this metal in plants across growing periods was 2.07 mg kg^−1^DM. Compared to the results reported by Kotecki^34^ and Bosiacki et al.^39^, the content of Ni in the biomass of giant miscanthus was much lower. According to Bosiacki et al.^39^,miscanthus Ni concentration ranged from 5.29 to 5.73 mg kg^−1^DM,depending on the growing period. The highest bioaccumulation of Ni (an average from the years of research) was in response to the combinations of municipal sludge with mushroom substrate: SS_25_ + SMS_75_ and SS_75_ + SMS_25_, with 2.77 and 2.27 mgkg^−1^DM, respectively. On the other hand, the lowest value was recorded on the control plot (1.77 mg kg^−1^DM). On the remaining experimental units, the concentration of Ni in plants did not exceed 2 mg kg^−1^DM. Bosiacki et al.^39^ report higher amounts of Ni in control plants than in those treated with mineral nitrogen. Kalembasa and Malinowska^42^ observed that the harvest date affected the content of Ni in the aboveground parts of *Miscanthus* *sacchariflorus*. Additionally, plants harvested in summer contained more Ni than those harvested in winter. This chemical element is highly mobile in plants, and its excess can cause disorders of basic physiological processes such as photosynthesis and transpiration^29^.

**Table 5:** Concentration of Ni in *Miscanthus × giganteus* biomass (mg^.^kg^-1^ DM).

Years (B)	Experimental plots (A)						
	Control plot	SS	SS_75_ + SMS_25_	SS_50_ + SMS_50_	SS_25_ + SMS_75_	SMS	Mean
	Ni (mg kg^−1^DM)						
2018 (I)	2.03aB	2.89aA	3.01aA	2.96aA	3.08aA	1.89aB	2.64a
2019 (II)	1.78abBC	1.56bBC	2.02bB	1.16bC	3.22aA	2.03aB	1.96b
2020 (III)	1.51AbAB	1.12bB	1.77bAB	1.45bAB	2.01bA	1.77aAB	1.61b
Mean	1.77B	1.86B	2.27B	1.86B	2.77A	1.90B	2.07

Within a row, different uppercase letters indicate a significant difference, within a column, different lowercase letters indicate a significant difference.

SS—municipal sewage sludge dose introducing 170 kg N ha^−1^; SMS—mushroom substrate dose introducing 170 kg N ha^−1^; SS_75_ + SMS_25_; SS_50_ + SMS_50_; SS_25_ + SMS_75_—municipal sewage sludge used together with mushroom substrate in various proportions, each dose introducing 170 kg N ha^−1^.

Extraction of Zn from *Miscanthus × giganteus* biomass significantly varied across treatment combinations and years of research (Table 6). On average, the plants absorbed the most Zn, 61% and 36.5% more than on the control plot, in response to mushroom substrate (SMS) and municipal sewage sludge (SS) applied separately. Among all treatment combinations significantly the lowest Zn content was noted in giant miscanthus growing on the control plot. There were no significant differences in Zn accumulation between plants treated with SS_75_ + SMS_25_ and SS_50_ + SMS_50_ (sewage sludge and mushroom substrate combinations). The average extraction of Zn from plants growing on those plots did not differ much. The amounts of this metal accumulated by plants in subsequent years increased with yield and were the highest in the third one despite the lowest Zn content (Table 4). According to Kabata-Pendias and Pendias^29^, cereals and spinach (*Spinacia oleraceae*) are the most sensitive plants to Zn content in soil, and the greatest risk of toxicity is on sandy acidic soils. This was also confirmed by the reports of McBride^43^.

**Table 6:** Extraction of Zn in *Miscanthus × giganteus* biomass (mg ha^−1^).

Years (B)	Experimental plots (A)						
	Control plot	SS	SS_75_ + SMS_25_	SS_50_ + SMS_50_	SS_25_ + SMS_75_	SMS	Mean
2018 (I)	415.7bD	445.6cCD	444.9cD	657.8aB	495.1bC	883.7aA	602.2a
2019 (II)	448.9bD	606.1bB	516.0bC	334.3cE	751.5aA	739.9aA	566.1b
2020 (III)	577.8aCD	759.5aA	668.1aB	607.5bC	528.9bDE	511.4cDE	608.9a
Mean	442.2D	603.7B	543.0C	533.1C	579.8B	711.7A	568.9

Within a row, different uppercase letters indicate a significant difference, within a column, different lowercase letters indicate a significant difference.

SS—municipal sewage sludge dose introducing 170 kg Nha^−1^; SMS—mushroom substrate dose introducing 170 kg Nha^−1^; SS_75_ + SMS_25_; SS_50_ + SMS_50_; SS_25_ + SMS_75_—municipal sewage sludge used together with mushroom substrate in various proportions, each dose introducing 170 kg Nha^−1^.

Extraction of Ni was slightly different from that of Zn (Table 7). The highest value was noted in plants treated with SS_25_ + SMS_75_ and SS_75_ + SMS_25_,71% and 45% higher than on the control plot, where the lowest amounts were noted. On the plots with SS and SS_50_ + SMS_50_ the content of Ni in *Miscanthus × giganteus* yield did not differ statistically. The uptake of this metal with biomass significantly varied over growing periods, with the highest value in the third year and the lowest in the first. Jakubus^44^ found that nutrients in organic waste materials had a limited impact on the chemical composition of energy crops. According to Kalembasa and Malinowska^45^, due to the residual effect the intensity of metal uptake by *Miscanthus sacchariflorus* biomass increased in the third and partly in the fourth year after sewage sludge application. The authors report that sewage sludge application resulted in a threefold increase in Ni uptake, a twofold increase in Cr and Cu uptake and in a greater accumulation of Zn and Cd in the third year. Deans et al.^46^ found that heavy metal uptake was dependent on miscanthus maturity and was proportional to the soil content of heavy metals. According to some studies on dicotyledonous plants, the bioaccumulation of Zn by plants varies depending on its availability in soil and on growing conditions^47–50^.

**Table 7:** Extraction of Ni in *Miscanthus × giganteus* biomass (mgha^−1^).

Years (B)	Experimentalplots (A)						
	Control plot	SS	SS_75_ + SMS_25_	SS_50_ + SMS_50_	SS_25_ + SMS_75_	SMS	Mean
2018 (I)	18.88cE	28.18aD	30.58cC	32.77aB	35.88bA	20.54cE	27.81b
2019 (II)	20.83bE	24.18bD	32.56bB	12.52cE	48.69aA	29.27bC	28.01b
2020 (III)	30.98aD	24.91bE	39.26aB	30.64bD	36.28bC	44.92aA	34.50c
Mean	23.56E	25.76D	34.14B	25.31D	40.28A	31.58C	30.10

Within a row, different uppercase letters indicate a significant difference, within a column, different lowercase letters indicate a significant difference.

SS—municipal sewage sludge dose introducing 170 kg Nha^−1^; SMS—mushroom substrate dose introducing 170 kg N ha^−1^; SS_75_ + SMS_25_; SS_50_ + SMS_50_; SS_25_ + SMS_75_—municipal sewage sludge used together with mushroom substrate in various proportions, each dose introducing 170 kg Nha^−1^.

The content of Zn and Ni in *Miscanthus × giganteus* rhizomes after 3 years significantly varied depending on the residual effect of the waste materials (Table 8). It was the highest in response to the greatest dose of sewage sludge applied together with the smallest amount of mushroom substrate (SS_75_ + SMS_25_). Compared to other fertilized plots, the content of Zn in rhizomes was significantly lower after the application of SMS, probably because some amounts had been translocated to aboveground parts. According to Table 6, the amounts of this element in rhizomes from the SS_75_ + SMS_25_ plot was the highest of all treatment combinations. On the control plot, Zn content in miscanthus rhizomes was higher than on plots with SMS or with SS, by 38% and 15.4%, respectively (Table 8).

**Table 8:** Zn and Ni content in rhizomes, bioaccumulation coefficient and translocation factor in the third year of *Miscanthus × giganteus* cultivation.

Experimental plots	Content in rhizomes (mgkg^−1^DM)		BC of rhizomes		BC of aboveground parts		TF from rhizomes to aboveground parts	
	Zn	Ni	Zn	Ni	Zn	Ni	Zn	Ni
Control	64.1c	4.54d	0.449a	0.892b	0.198a	0.297a	0.440ab	0.333a
SS	54.2d	8.55b	0.166d	0.904b	0.105b	0.118c	0.630a	0.131f
SS_75_ + SMS_25_	82.7a	10.11a	0.272b	1.209a	0.099b	0.212b	0.364b	0.175e
SS_50_ + SMS_50_	60.7c	6.14c	0.194c	0.525d	0.092c	0.124c	0.474ab	0.236c
SS_25_ + SMS_75_	78.4b	9.91a	0.273b	1.190a	0.102b	0.241b	0.374ab	0.203d
SMS	39.7e	5.97c	0.146e	0.740c	0.074d	0.219b	0.508ab	0.296b
Mean	63.3	7.54	0.232	0.887	0.104	0.189	0.465	0.229

Within a column, different lowercase letters indicate a significant difference.

SS—municipal sewage sludge dose introducing 170 kg Nha^−1^; SMS—mushroom substrate dose introducing 170 kg Nha^−1^; SS_75_ + SMS_25_; SS_50_ + SMS_50_; SS_25_ + SMS_75_—municipal sewage sludge used together with mushroom substrate in various proportions, each dose introducing 170 kg Nha^−1^.

The lowest content of Ni was in rhizomes from the control plot, almost two times lower than on plots with SS_75_ + SMS_25_, SS_25_ + SMS_75_ and SS. After the third year the average amounts of Ni in rhizomes, with 7.54 mg kg^−1^DM, were over three times higher than in aboveground parts, with 2.07 mg kg^−1^DM (Table 5). Rhizomes, on average, also accumulated more Zn (63.3 mg kg^−1^DM) than aboveground parts (40.52 mg  kg^−1^DM), but the difference was not as great as for Ni. In the third year a much higher bioaccumulation coefficient (BC) of the heavy metals, especially of Ni, was found in giant miscanthus rhizomes than in aboveground parts (Table 8). The average BC value of Ni in rhizomes was 0.887whereas in aboveground parts it was 0.189. In the case of Zn it was 0.232 in rhizomes and 0.104 in shoots and leaves. The above values meant that waste materials used in the experiment did not pose a threat to the environment. On this basis, it can be assumed that the root system was a serious barrier to the movement of these elements to aboveground parts. The accumulation of metals in rhizomes and aboveground parts was dependent primarily on the type of metal and, to a lesser extent, on the residual effect of waste materials. This was confirmed by Kloke et al.^51^, who reported that bioaccumulation coefficients in the soil–plant system for Cd and Zn ranged from 1 to 10, while for Pb from 0.01 to 0.1.

The translocation factor (TF) is used to determine heavy metal movement from plant roots to aboveground parts. Based on heavy metal concentration in the plant, its phytoextraction capacity can be estimated. According to statistical calculations, the residual effect of the organic waste materials had a significant impact on the translocation factor in the rhizome-aboveground system of giant miscanthus in the third year (Table 8). The Ni translocation factor was significantly the highest on the control plot (0.333). In the case of Zn, the highest TF value (0.630) was recorded on plots with sewage sludge (SS). Mobility of heavy metals in soil and plants and their bioaccumulation depend primarily on soil and environmental conditions, plant species and the type of metal^52^. Ociepa et al.^48^ noted a much higher translocation of Zn from prairie cordgrass (*Spartina pectinata*) roots to aboveground parts than in the case of Pb. Their accumulation in individual parts of the grass was dependent mainly on the type of metal.

**Figure 2:**
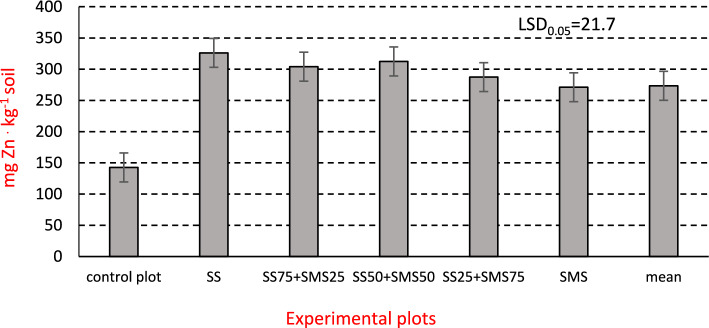
Soil Zn content in the third year of *Miscanthus × giganteus* cultivation (mgkg^−1^DM soil).

**Figure 3:**
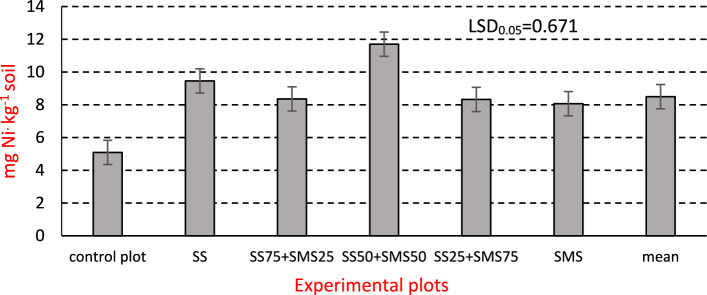
Soil Ni content in the third year of *Miscanthus × giganteus* cultivation (mgkg^−1^DM soil).

Compared to the first year, at the end of the third one increased content of Zn and Ni was noted in the soil to which waste materials were applied (Figs. 2 and 3, Table 1). In soil from the control plot, their content was significantly the lowest. The highest amounts of Zn were recorded in soil treated with municipal sewage sludge (SS), and in the case of Ni with sewage sludge applied together with mushroom substrate (SS_50_ + SMS_50_). Malinowska^53^ observed that soil Zn concentration increased even twice after the application of sewage sludge, and the increase was dependent on the dose. After the third year of miscanthus cultivation, soil content of both heavy metals was significantly higher on plots with sewage sludge (SS) than on those with mushroom substrate (SMS): by 20.3% in the case of Zn and by 17.2% in the case of Ni. This was due to the difference in the chemical composition of the organic wastes. In Poland, according to the Regulation of the Ministry of the Environment^54^, the content of Zn and Ni in agricultural soils designated as subgroup II-2 (i.e. light mineral soils with pH above 6.5) should not exceed 500 mg Zn kg^−1^ and 150 mg Nikg^−1^. In the present experiment their soil content after the third year of *Miscanthus x giganteus* cultivation was much lower.

A statistically significant negative correlation (r = − 0.875) was noted between Zn content in soil and BC of giant miscanthus aboveground parts (Table 9). In turn, between Zn content in soil and BC of rhizomes a significant positive correlation of r = 0.843 was noted. As regards the relationship between the Zn translocation factor (from rhizomes to aboveground parts of giant miscanthus) and Zn content in the soil, a weak positive correlation was found, not statistically significant. The content of Ni in rhizomes was significantly positively correlated with its content in the soil, with r = 0.985 (Table 10). A significant negative relationship was found between the translocation factor of Ni (from rhizomes to aboveground parts)and its content in soil, with r = − 0.832.

**Table 9:** Linear correlation coefficient between Zn content in soil, in aboveground parts, in rhizomes, uptake with biomass yield, bioaccumulation coefficient and translocation factor (all arranged in pairs).

Zn	Zn content in soil	Zn content in aboveground parts	Zn uptake with biomass yield	Zn content in rhizomes	BC of aboveground parts	BC of rhizomes	TF, from rhizomes to aboveground parts
Zn content in soil	1.00						
Zn content in aboveground parts	0.278	1.00					
Zn uptake with biomass yield	0.184	0.527	1.00				
Zn content in rhizomes	0.032	0.519	0.559	1.00			
BC of aboveground parts	− 0.875*	0.217	0.100	0.196	1.00		
BC of rhizomes	0.843*	0.147	0.191	0.489	0.917*	1.00	
TF, from rhizomes to aboveground parts	0.238	0.154	− 0.252	− 0.758	− 0.135	− 0.516	1.00

*Significant relationship p ≤ 0.05.

**Table 10:** Linear correlation coefficient between Ni content in soil, in aboveground parts, in rhizomes, uptake with biomass yield, bioaccumulation coefficient and translocation factor (all arranged in pairs).

Ni	Ni content in soil	Ni content in aboveground parts	Ni uptake with biomass yield	Ni content in rhizomes	BC of aboveground parts	BC of rhizomes	TF, from rhizomes to aboveground parts
Zn content in soil	1.00						
Ni content in aboveground parts	0.316	1.00					
Ni uptake with biomass yield	0.077	0.793	1.00				
Ni content in rhizomes	0.985*	0.316	0.077	1.00			
BC of aboveground parts	− 0.211	0.592	0.432	− 0.211	1.00		
BC of rhizomes	0.753	0.485	0.188	0.753	0.437	1.00	
TF, from rhizomes to aboveground parts	− 0.832*	0.239	0.350	− 0.832*	0.654	− 0.391	1.00

*Significant relationship p ≤ 0.05.

Finally, it should be emphasized that bioaccumulation of chemical elements in the soil–plant system and their movement to individual parts of plants depend primarily on soil conditions and on the metal. For each heavy metal the correlation between its content in the soil and in the plant was different and so were BC and TF coefficients. On the basis of the results it should be concluded that *Miscanthus × giganteus* rhizomes constitute a barrier that stops Zn and Ni movement, which is an extremely positive observation, making miscanthus biomass a very useful energy resource.

## Conclusions

The yield of *Miscanthus × giganteus* fresh and dry matter significantly varied across treatment combinations and growing seasons. The highest average dry matter yield was noted in response to the application of mushroom substrate either on its own(SMS)or in combination with municipal sewage sludge (SS_75_ + SMS_25_) and the lowest on the control plot. In the third year of cultivation dry matter yield was the highest with 21.58 Mg kg^−1^. As an average of all treatment combinations, the highest but still moderate content of Zn and Ni in the aboveground parts of giant miscanthus was in the first year of the study. The uptake of the heavy metals by *Miscanthus × giganteus* significantly varied across treatment combinations and growing periods. The highest accumulation of Zn was recorded in plants treated with SMS and SS, and of Ni on plots with SS_25_ + SMS_75_ and SS_75_ + SMS_25_. At the end of the third growing period, giant miscanthus rhizomes contained much higher amounts of Zn and Ni than aboveground parts. Bioaccumulation of heavy metals in energy crops may contribute to the emergence of new environmental problems. Because of residues from combustion processes of energy crops with considerable quantities of heavy metals, plants grown on soil treated with organic waste may be classified as hazardous for the environment. However, despite soil treatment with municipal sewage sludge, in the present experiment biomass did not pose a threat in terms of its content of selected heavy metals. Compared to the first year, in the third one, an increased soil content of Zn and Ni was found, significantly higher on the plot with municipal sewage sludge (SS) than on that with mushroom substrate (SMS).

Sewage sludge can contaminate the soil and cause excessive accumulation of heavy metals in the plant. Therefore, it is necessary to strictly comply with the legal regulations limiting its use and to select such plants as *Miscanthus × giganteus*, accumulating heavy metals in the underground part.

## Data Availability

The datasets generated and analysed during the current study are not publicly available as they are the authors’ own data, but they are available from the corresponding author on reasonable request.
